# Bilateral Acute Optic Perineuritis Associated With COVID-19 in a Patient With Seronegative Myelin Oligodendrocyte Glycoprotein (MOG) Antibody

**DOI:** 10.7759/cureus.18234

**Published:** 2021-09-24

**Authors:** Liaquat Ali, Muhammad Naeem, Beatriz Canibano, Anju John, Ambreen Iqrar

**Affiliations:** 1 Neurology, Hamad General Hospital, Doha, QAT; 2 Neurology, Weill Cornell Medicine-Qatar, Doha, QAT; 3 Medicine, Hamad General Hospital, Doha, QAT; 4 Internal Medicine, Hamad General Hospital, Doha, QAT

**Keywords:** angiotensin-convering-enzyme-2 (ace-2), neuromyelitis optica spectrum disorder (nmosd), myelin oligodendrocyte glycoprotein (mog), anterior ischemic optic neuropathy (aion), severe acute respiratory syndrome coronavirus-2 (sars-cov-2)

## Abstract

Severe acute respiratory syndrome coronavirus-2 (SARS-Cov-2) may cause various neuro-ophthalmologic manifestations including optic perineuritis. Optic perineuritis is a rare form of orbital inflammatory disease in which optic nerve sheath is inflamed and nonspecific fibrotic thickening with classic radiological finding is a perineural enhancement of optic nerve sheath.

A 45-year-old gentleman with known diabetes mellitus, hypertension and dyslipidemia was admitted with a critically ill COVID-19 infection. During the recovery period, the patient developed sudden onset of painless loss of vision. MRI head and orbit with gadolinium was suggestive of optic perineuritis. Other secondary causes of autoimmune or vasculitis myelin oligodendrocyte glycoprotein (MOG) antibody disease and other common central nervous system (CNS) infection were excluded. The patient had dramatic response with steroids.

This is the first rare case report of COVID-19-related optic perineuritis in critically ill COVID-19 patients with seronegative MOG antibody. Optic perineuritis is a rare orbital inflammatory disease and underlying mechanisms may arise from systemic response to COVID-19 infection as well as direct effects of the virus via angiotensin-converting enzyme 2 (ACE-2) receptors on ocular tissues. Optic perineuritis is a rare disease with inflammation restricted to the optic nerve sheath. Neuroimaging of the brain and orbit is the most important modality of choice for visualizing optic nerve sheath and optic nerve. Delay in the diagnosis of COVID-19-related optic perineuritis, may result in permanent optic nerve injury and irreversible vision loss.

## Introduction

Severe acute respiratory syndrome coronavirus-2 (SARS-Cov-2) is a new genus of beta coronavirus that may cause visual impairment due to dacryoadenitis, conjunctivitis, tonic pupils, vitritis, central retinal artery or venous occlusion, retinitis, retinal bleeding, panuveitis, anterior ischemic optic neuropathy (AION), optic perineuritis, optic neuritis and occipital ischemic stroke [1].

The optic nerve carries greater than one million axons that derive from retinal ganglion cells and are projected to the visual nuclei [2]. An optic nerve lesion produces usually monocular visual loss; others include dyschromatopsia, pain especially with eye movement and papillitis. The potential causes of optic neuropathy are diverse such as ischemic optic neuropathy, optic neuritis (demyelinating diseases), inflammatory or infectious optic neuropathy, sarcoidosis, neoplasms (compressive or infiltrative), toxic or metabolic, traumatic and genetic disorders (Leber's hereditary optic neuropathy) etc. Ischemic optic neuropathy is the most common etiology in older patients (> 50 years) while optic neuritis is in younger adults [3].

Edmunds and Lawford in 1883 first described two forms of optic perineuritis (exudative and purulent) in the histopathologic specimen of optic nerve showing inflammatory infiltrates organized around the nerve [4]. Optic perineuritis (or peri-optic neuritis) is a rare form of orbital inflammatory disease in which optic nerve sheath is inflamed, resulting in mark nonspecific fibrotic thickening in contrast to optic neuritis resulting in inflammation of optic nerve axons [5]. Visual loss in optic perineuritis has been attributed to secondary ischemic infarction of optic nerve due to circumferential compression of nerve periphery by mass of thickened optic nerve sheath [6]. Unlike isolated optic neuritis, optic perineuritis may present with orbital signs and symptoms (ophthalmoplegia, ptosis and exophthalmos) [7]. Optic perineuritis is usually idiopathic (primary) but can be secondary to systemic diseases such as autoimmune diseases, myelin oligodendrocyte glycoprotein (MOG) antibody-associated optic neuropathy, IgG4-related disease, para-infectious, primary or metastatic optic nerve neoplasms, etc. Previously, optic perineuritis was defined as an optic neuropathy with optic disc edema but without optic nerve dysfunction and with normal intracranial pressure. However, with the advance of neuroimaging techniques (thin orbital cuts MRI with fat-suppressed T1WI with gadolinium) that allowed dedicated imaging of optic nerve sheath, the term optic perineuritis was redefined [8].

Optic perineuritis is clinically diagnosed and confirmed on the classical radiological finding of perineural enhancement of optic nerve sheath in gadolinium T1WI fat-suppressed MRI sequence that appears as tram track in axial view and doughnut in coronal view [9]. Other routine laboratory tests include serology for syphilis, serum angiotensin-converting enzyme (ACE), Mantoux test or interferon-gamma release assay (IGRA), chest X-ray for tuberculosis, anti-nuclear antibodies (ANA), anti-neutrophil cytoplasmic autoantibodies (ANCA), IgG4 level, C-reactive protein (CRP), erythrocyte sedimentation rate (ESR), lumbar puncture, etc. The management of optic perineuritis is corticosteroid treatment with dramatic improvement [10].

## Case presentation

A 45-year-old gentleman with a known case of diabetes mellitus, hypertension and dyslipidemia was admitted with complaints of fever, headache, cough and dyspnea. Nasal and oropharyngeal swab for the real-time polymerase chain reaction (RT-PCR) test for SARS-CoV-2 came out positive. Chest X-ray showed accentuated bronchovascular and perihilar marking with multifocal patchy ground-glass opacities in the bilateral lung field. He was hospitalized with severe COVID-19 pneumonia; his respiratory status deteriorated rapidly for 3 days since admission and required high oxygen and intubated ventilation due to respiratory distress. During his ICU course he developed sepsis and required broad-spectrum antibiotics. He had acute kidney injury for which he required one session of dialysis and improved with medical treatment. He started per rectal bleeding and was managed conservatively with input from the gastroenterology team. He had high troponin and the cardiology team was consulted and their opinion was of type 2 myocardial infarction secondary to sepsis and managed conservatively. Echocardiography showed a normal ejection fraction of 56% without any left ventricle clot. He was successfully extubated after 14 days. He developed critical illness myopathy after the prolonged ICU course.

**Figure 1:**
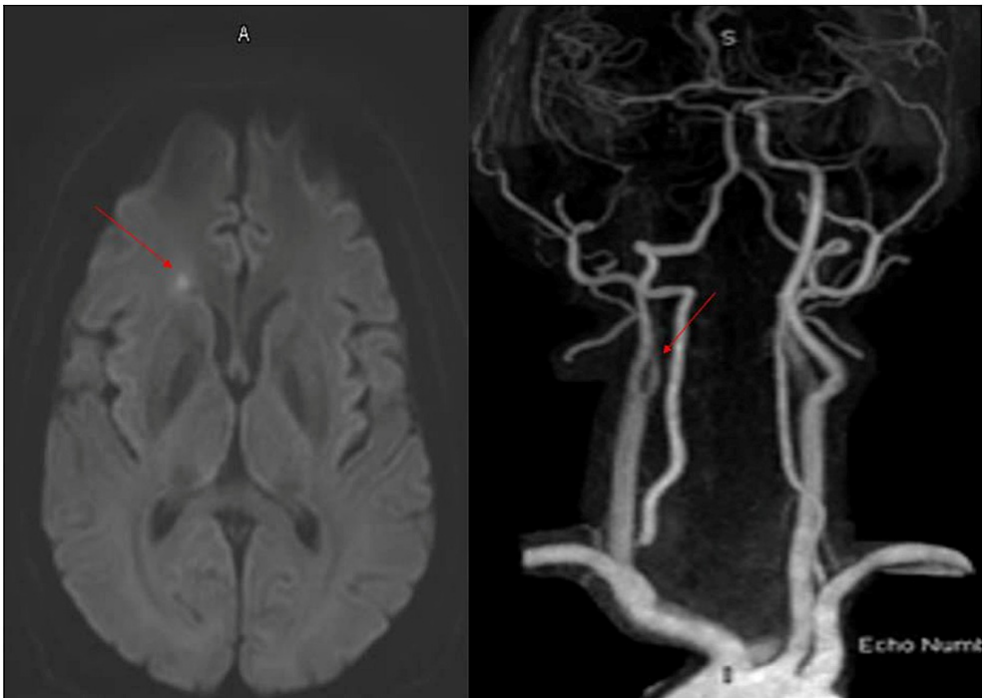
MRI head diffusion-weighted image (DWI) and MRA head and neck vessels with gadolinium MRI head DWI sequence showed a small right deep frontal white matter region acute ischemic stroke (as shown by the red arrow in the left panel). MRA (magnetic resonance angiography) head and neck vessels with gadolinium showed tapering of the right internal carotid artery (ICA) just after carotid bifurcation and proximal segment of left internal carotid artery followed by distal occlusion along the rest of its neck and intracranial segment (as shown by the red arrow in the right panel).

After one month's stay in the intensive care unit (ICU) with recovering from critical COVID-19 infection, the patient developed of sudden onset of bilateral painless loss of vision for 1 day. On examination right eye had perception of light only and the left eye with the counting of fingers at 1 meter with normal intraocular pressure. Right fundus showed severe non-proliferative diabetic retinopathy (NPDR), Weiss ring floaters, peripheral flat retinal break and peripheral degeneration whereas the left eye with severe NPDR, peripheral degeneration which could not explain his sudden vision loss. MRI head diffusion-weighted image (DWI) showed a tiny right deep frontal acute ischemic stroke (as shown in Figure 1). Magnetic resonance angiography (MRA) head and neck vessels with gadolinium showed tapering of the proximal segment of the left internal carotid artery after carotid bifurcation followed by distal occlusion with absent flow and refilling of the terminal segment through reflex circulation (as shown in Figure 1).

**Figure 2:**
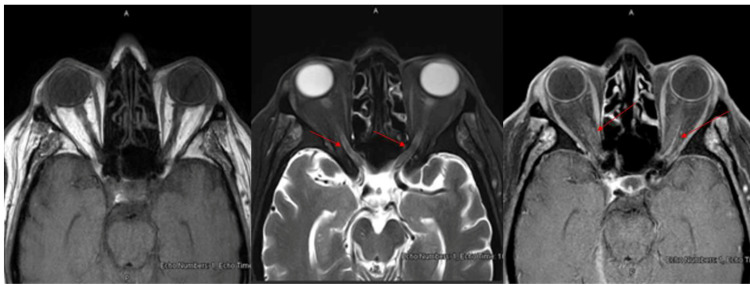
MRI orbits with gadolinium axial view (T1WI, T2WI, T1WI-Gad.) MRI orbits with gadolinium with axial view showed distal intra-orbital optic nerve sheath faint enhancement without extension to proximal intra-orbital or pre-chiasmatic optic nerve segment, normal appearance of the uveoscleral coat and extraocular muscles (as shown by the red arrows). T1-weighted image (T1WI), T2-weighted image (T2WI), T1WI with gadolinium (T1W1Gad.)

MRI orbits with gadolinium axial view of T1-weighted image (T1WI), T2-weighted image (T2WI), T1WI with gadolinium showed distal intra-orbital optic nerve sheath faint enhancement (as shown by red arrows in Figure 2), however, no extension into pre-chiasmatic or proximal intra-orbital segments and normal appearance of the uveoscleral coat and normal muscle cone with no intra- or extra-conal abnormality (as shown by red arrows in Figure 2).

**Figure 3:**
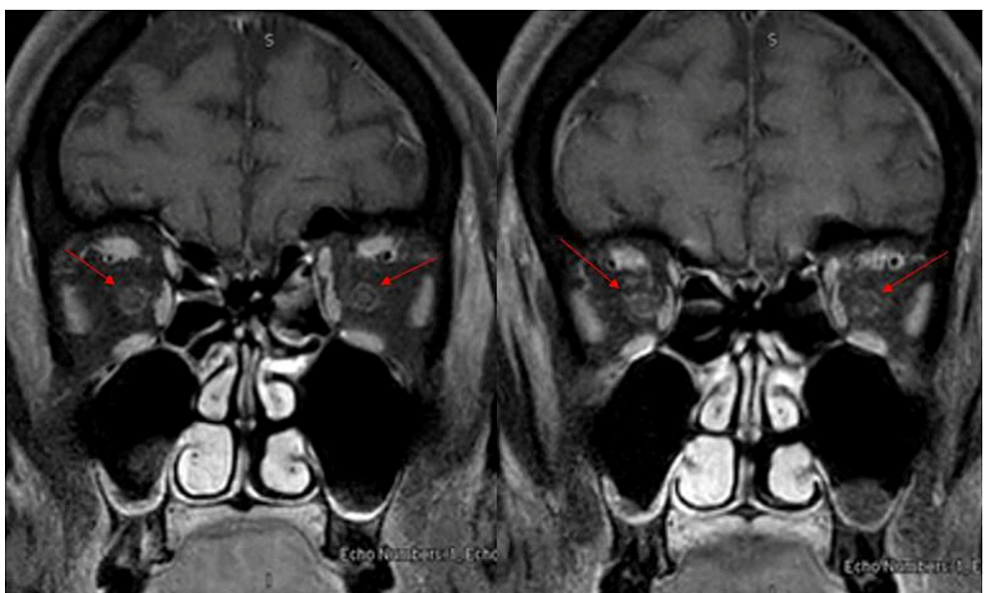
MRI orbits T1-weighted image with gadolinium MRI orbits T1-weighted image with gadolinium fat-suppressed coronal view showed distal intra-orbital optic nerve sheath faint enhancement and appearance of doughnuts like shape (as shown by red arrows).

MRI orbits fat-suppressed T1-weighted image with gadolinium coronal view showed distal intra-orbital optic nerve sheath faint enhancement and appearance of doughnuts like shape and normal appearance of the uveoscleral coat and extraocular muscles (as shown by red arrows in Figure 3).

MRI cervical and thoracic spinal cord with gadolinium was unremarkable for any detectable intermedullary signal abnormality. Doppler ultrasound carotid artery showed (0.4x 0.2 cm) calcified thrombus in the proximal right internal carotid artery just at the level of the bifurcation with turbulent flow with no significant stenosis and good distal run-off. In the left proximal internal carotid artery (ICA), an echogenic thrombus (0.4 x 0.2 cm) with no significant stenosis was detected and normal arterial flow with good distal run-off.

Routine laboratory results showed high ferritin, CRP, lactate dehydrogenase (LDH), IL-6, urea, creatinine, troponin- T, N-terminal pro B-type natriuretic peptide (NT-pro BNP), high creatine kinase (CK), alanine transaminase (ALT), aspartate transaminase (AST), high glycated hemoglobin (HbA1c), normal thyroid-stimulating hormone (TSH), vitamin B12, negative antinuclear antibody (ANA), negative antineutrophil cytoplasmic autoantibody (ANCA) and negative anticyclic citrullinated peptide antibody. Lumbar puncture showed slightly high protein with normal white cell count. Patient had negative CSF oligoclonal bands, negative for myelin oligodendrocyte glycoprotein (MOG-IgG) antibody. Viral meningitis PCR panel including herpes simplex virus (HSV) 1, 2 was negative and no growth of CSF cultures and autoimmune vasculitis panel were negative (as shown in Table 1).

**Table 1:** Blood, Biochemical and CSF lab test Table 1 shows very high COVID-19 inflammatory markers (Ferritin, CRP, LDH, IL-6), high urea, creatinine, high troponin, high NT-pro BNP,  high CK, high ALT, AST liver enzymes, high HbA1c , normal TSH, vitamin B12, negative ANA, negative ANCA and negative anti-CCP antibody. Lumbar puncture showed slightly high protein with normal white cell count. Patient had negative CSF oligoclonal bands, negative for MOG-IgG antibody. Viral meningitis PCR panel included HSV 1, 2 were negative and no growth of CSF cultures. CRP: C-reactive protein; LDH: lactate dehydrogenase; HbA1c: glycated hemoglobin; ALT: alanine aminotransferase; AST: aspartate aminotransferase; CPK: creatine phosphokinase; NT-pro BNP: N- terminal pro B-type natriuretic peptide; C3: complement component 3; C4: complement component 4; ANCA: antineutrophil cytoplasmic autoantibody; DAT: direct antiglobulin test; CSF: cerebrospinal fluid; PCR: polymerase chain reaction; HHV: human herpesvirus; ANA: antinuclear antibody; CMV: cytomegalovirus; TSH: thyroid-stimulating hormone; MOG: myelin oligodendrocyte glycoprotein; HSV: herpes simplex virus; CCP: cyclic citrullinated peptide; VSV: varicella-zoster virus.

Indicators	Highest values	Indicators	Highest values
CRP	249	Ferritin	1267
LDH	512	IL-6	293
HbA1c	12.3%	TSH	2.5
Vit B12	410	Folate	21
ALT	204	AST	213
Urea	47.6	Creatinine	316
CPK	13260	Troponin-T HS	331
NT-pro BNP	3444	C3	1.56
C4	0.52	Anti-CCP	Negative
ANCA	negative	ANA	Negative
DAT	positive	COVID-19 PCR	positive (CT value=18)
CSF WBC	1	CSF RBC	0
CSF Protein	0.74	CSF sugar	10
CSF IgG	69 (High)	CSF Albumin	479 (High)
CSF Oliogoclonal	negative	IgG index	0.6
CMV PCR	Negative	HSV 1,2 PCR	Negative
HHV PCR	Negative	VZV PCR	Negative
Entrovirus PCR	Negative	Anti-MOG IgG	Negative

After excluding secondary etiologies, finally, the patient was diagnosed to have bilateral optic nerve perineuritis probably due to COVID-19-related inflammatory changes. He was started on IV methylprednisolone pulse therapy for 3 days and he showed dramatic response within a week. His vision improved to counting fingers at 1 meter on the right eye and complete recovery of the left eye. Hence, he continued a steroid course orally of 60mg daily for 2 weeks and after slowly tapered weekly with a 10mg dose. He was transferred to the rehabilitation center for the critical illness myopathy physical therapy and follow-up neuro-ophthalmology clinic after 3 months.

## Discussion

This is the first rare case report of COVID-19-related bilateral acute optic perineuritis in a critically ill COVID-19 patient from a tertiary care hospital in Doha, Qatar. Optic perineuritis is an orbital inflammatory disease in which the optic nerve sheath is inflamed, result in a nonspecific fibrotic thickening. Optic perineuritis is usually idiopathic but can be secondary to systemic diseases. Optic perineuritis is radiologically diagnosed based on classic findings of perineural enhancement of optic nerve sheath that appears as tram tracks in axial and doughnuts in coronal view. In patients with MOG-associated antibody disease, the bilateral optic nerve is more commonly affected than in neuromyelitis optica spectrum disorder (NMOSD) or multiple sclerosis. By exclusion of negative MOG Abs, negative autoimmune or vasculitis work-up, CSF cultures showed no growth, negative metabolic results and dramatic improvement by steroids, we believed that this patient bilateral acute optic perineuritis may be related to COVID-19 infection. 

The underlying mechanisms of neuro-ophthalmological complications of COVID-19 may arise from systemic response to COVID-19 infection as well as direct effects of the virus. The mechanism of uptake of SARS-CoV-2 into neural and ocular tissue has not been well-elucidated but one of the hypotheses is the presence of ACE-2 receptors on neural and ocular tissues; SARS-CoV-2 binds to ACE-2, a membrane-bounded protein, as its point of entry into cells, allowing SARS-CoV-2 to infiltrate specific tissues in the body, resulting in a diverse range of complications [11]. Another hypothesis is the direct inflammation of nerves, results in myelin sheath damage as this have been seen when mice were inoculated with mouse hepatitis virus (MHV) A56 and isolated optic nerve sheath were infiltrated with inflammatory cells [12]. Other authors suggested optic nerve ischemia secondary to the hypercoagulable state as one of the possible mechanisms of COVID-19-associated coagulopathy. Critically ill patients with COVID-19 develop signs of severe systemic inflammation consistent with a cytokine release syndrome-like presentation and elevated proinflammatory cytokines [13]. A proinflammatory state and complement activation may be associated with thrombophilia [14] and thrombotic microvascular injury in patients with severe COVID-19 [15].

In the review of present literature on optic perineuritis associated with COVID-19 infection and vaccination, there were two case reports, the first was associated with seropositive MOG antibody and the other perinuclear anti-neutrophil cytoplasmic antibodies (P-ANCA)-associated post BNT162B2 mRNA COVID-19 vaccination, suspected of underlying autoimmune mechanism and responded effectively to immunotherapy.

## Conclusions

Neuro-ophthalmic manifestations of COVID-19 are uncommon; nevertheless, they may cause optic perineuritis leading to permanent visual impairment and blindness. Optic perineuritis is a rare disease presenting with inflammation restricted to the optic nerve sheath. MRI brain and orbit with gadolinium fat-suppressed T1-weighted images are the most important modality of choice for visualizing optic nerve sheath and optic nerve. Delay in the diagnosis and treatment of COVID-19-associated optic perineuritis may result in permanent optic nerve injury and irreversible vision loss. This case report help physicians in the early evaluation and treatment of COVID-19-associated optic perineuritis and other neuro-ophthalmic manifestations of COVID-19. It requires future prospective cohort studies.
